# What factors control superficial lava dome explosivity?

**DOI:** 10.1038/srep14551

**Published:** 2015-09-30

**Authors:** Georges Boudon, Hélène Balcone-Boissard, Benoît Villemant, Daniel J. Morgan

**Affiliations:** 1 Institut de Physique du Globe de Paris, Sorbonne Paris Cité, Univ. Paris Diderot, CNRS, F-75005, Paris, France; 2 Sorbonne Universités, UPMC Univ Paris 06, CNRS, Institut des Sciences de la Terre de Paris (iSTeP), 4 place Jussieu 75005 Paris, France; 3 Institute of Geophysics and Tectonics, School of Earth & Environment, University of Leeds, Leeds, LS2 9JT UK

## Abstract

Dome-forming eruption is a frequent eruptive style and a major hazard on numerous volcanoes worldwide. Lava domes are built by slow extrusion of degassed, viscous magma and may be destroyed by gravitational collapse or explosion. The triggering of lava dome explosions is poorly understood: here we propose a new model of superficial lava-dome explosivity based upon a textural and geochemical study (vesicularity, microcrystallinity, cristobalite distribution, residual water contents, crystal transit times) of clasts produced by key eruptions. Superficial explosion of a growing lava dome may be promoted through porosity reduction caused by both vesicle flattening due to gas escape and syn-eruptive cristobalite precipitation. Both processes generate an impermeable and rigid carapace allowing overpressurisation of the inner parts of the lava dome by the rapid input of vesiculated magma batches. The relative thickness of the cristobalite-rich carapace is an inverse function of the external lava dome surface area. Explosive activity is thus more likely to occur at the onset of lava dome extrusion, in agreement with observations, as the likelihood of superficial lava dome explosions depends inversely on lava dome volume. This new result is of interest for the whole volcanological community and for risk management.

Dome-forming eruption is a frequent eruptive style^1^; lava domes result from continuous or episodic slow extrusion of viscous lava. Most dome-forming eruptions produce highly microcrystallized and highly- to almost totally-degassed magmas which have a low explosive potential. During lava dome growth, recurrent collapses of unstable parts are the main destructive process of the lava dome, generating block- and ash-flows (concentrated pyroclastic flows) hereafter referred to as “concentrated pyroclastic density currents” (C-PDC)^2^. C-PDC’s are channelized in valleys^3^,^4^,^5^ and have a high, but localized, damage potential that largely depends on the collapsed volume^6^. Sometimes, a dilute ash cloud surge develops at the top of the concentrated flow with an increased destructive effect, because it may overflow ridges and affect larger areas^5^. In addition, large lava dome collapses can induce a depressurization of volatile-bearing magma within the conduit, leading to vulcanian explosions^3^,^5^. By contrast, violent, laterally-directed explosions may occur at the base of a growing lava dome: these generate dilute and turbulent pyroclastic flows, or surges (péléean ‘nuées ardentes’^7^) hereafter referred to as “dilute pyroclastic density currents” (D-PDC)^2^. They have a high velocity and their propagation is poorly dependent on the topography, leading to highly destructive effects^7^,^8^. Numerous studies on lava dome behaviors exist^6^,^9^,^10^,^11^,^12^,^13^, but the triggering of lava dome explosions—an important topic in hazard mitigation and risk assessment^14^—is poorly understood, which this article hopes to address.

A series of dome-forming eruptions are investigated through the petrology of their volcanic products. At Montagne Pelée (Martinique), during the first months of the 1902–1905 eruption, violent, superficial, laterally-directed explosions at the base of the growing lava dome generated D-PDC’s which devastated the south-western flank of the volcano^7^,^8^. The May, 8^th^ 1902 event, which occurred two days after the beginning of lava dome growth, was responsible for the death of 28 000 inhabitants. It was the first of a series of 7 eruptions, ending on August, 30^th^ 1902. Following this explosive phase, the 1902–1905 activity moved toward a lava dome collapse style producing C-PDC’s^7^. This eruptive style persisted throughout the 1929–1932 eruption^15^. The P1 eruption (650 y. BP), also started by the growth of a lava dome that was destroyed, as for the 1902 eruption, by two violent, superficial, laterally-directed explosions initiated at the base of the growing lava dome^16^. They generated D-PDC’s which devastated the south-western flank of the volcano. This first phase was followed by a plinian phase, generating pumice fallout that covers the volcano flanks. The May, 8^th^ 1902 D-PDC, one of the D-PDC from the P1 eruption and two C-PDC deposits from the 1929-1932 eruption as well as the two 1902–1905 and 1929–1932 lava domes were sampled.

During the on-going eruption of Soufrière Hills (Montserrat), a lava dome has been permanently present since 1995, alternating periods of quiescence with periods of lava dome growth and recurrent destruction^5^. Destruction occurs by collapses of variable volumes with attendant vulcanian explosions, together generating C-PDC^5^. This eruption destroyed the Castle Peak lava dome previously emplaced (1650 AD;^17^). The Castle Peak lava dome and a series of lava domes of the 1996–1997 period were sampled with their associated C-PDC deposits.

At Santa Maria-Santiaguito volcano (Guatemala), a plinian eruption occurred in 1902 and was followed 20 years later by a dome-forming eruption that is still on-going. A series of lava domes were built and produced more-or-less voluminous C-PDC channeled in the main valleys of the south-western flank of the volcano^18^,^19^. Different lava domes (Caliente: 1922–1925, El Monje: 1950’s, El Brujo: 1970’s), lava flows (1971–1972 and 1973–1975) and the C-PDC deposit from the large Caliente lava dome collapse of 1929 were sampled.

In La Chaîne des Puys (Massif Central, France), the Puy de Dôme eruption (~10,800 y. BP)^20^ only produced C-PDCs whereas the Puy Chopine eruption (~9,700 y. BP)^20^ generated a D-PDC during its first phase of activity, destroying the emerging lava dome. The lava dome and the C-PDC deposit from Puy de Dôme and the D-PDC deposit from Puy Chopine were sampled.

Finally, a sample of the lava dome from the 1991–1994 dome forming eruption of Unzen (Japan) is included in this study for comparison.

Petrological data from the plinian phase of the P1 eruption (Montagne Pelée), the 1902 plinian eruption of Santa Maria^21^,^22^ and the 1997 vulcanian explosions of Soufrière Hills^23^ are also included for comparison of plinian or vulcanian eruptions with dome-forming eruptions at the same volcanoes during the active periods studied.

## Results

### Magma composition

Magmas of Montagne Pelée and Montserrat are acid andesites (60–62 and 58–60 wt% SiO_2_ respectively), those of Santiaguito are dacites (65–67 wt% SiO_2_) and those of La Chaîne des Puys are trachytes (66–69 wt% SiO_2_). All residual glasses are rhyolitic (70–79 wt% SiO_2_).

### Textural characteristics: density, vesicularity, cristobalite content

**Figure 1:**
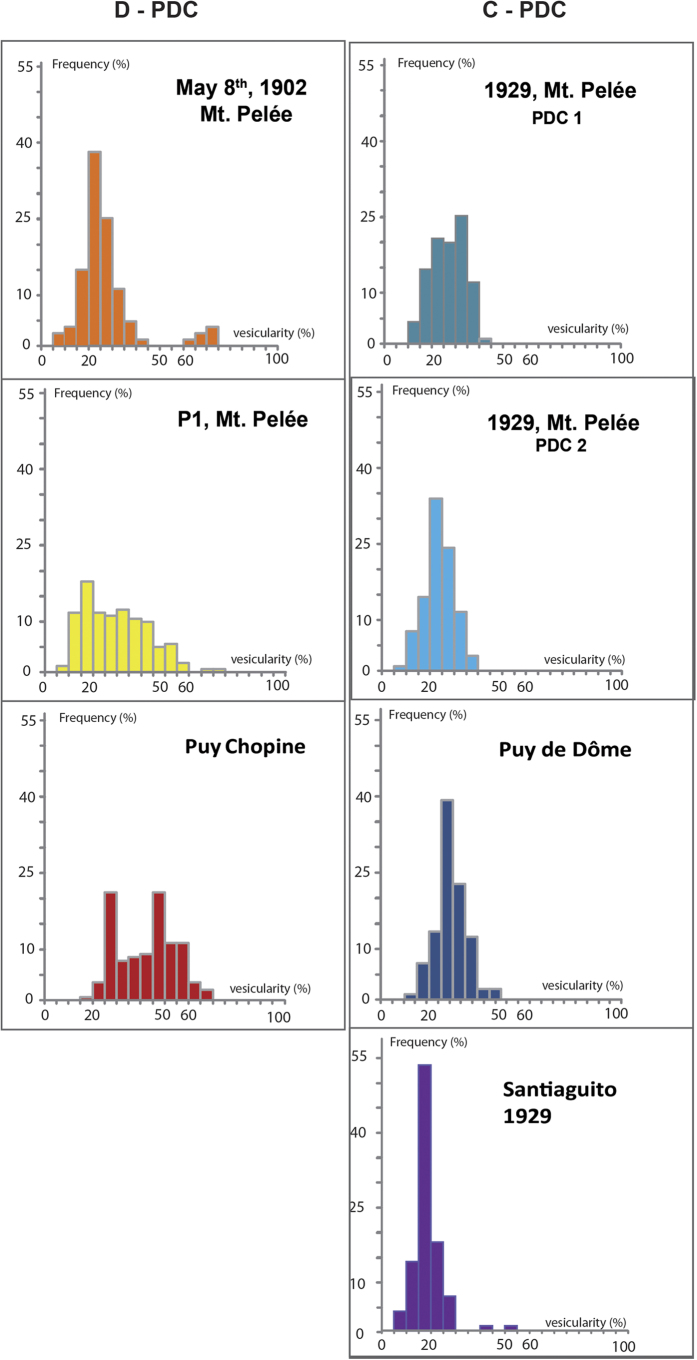
Bulk rock vesicularity distribution (frequency %) of samples from Pyroclastic Density Current (PDC) deposits, as a function of vesicularity (in volume %). The distribution is based on at least 100 clasts for each deposit in the fraction 16–32 mm, The left column refers to D-PDCs (Montagne Pelée : May 8^th^, 1902, lava dome phase of P1 eruption; Puy Chopine). The right column refers to C-PDCs (Montagne Pelée, 2 C-PDCs from the 1929–1932 eruption; Puy de Dôme; Santiaguito, 1929). The C-PDC vesicularity distribution is unimodal for the four examples (centered around 27% and 22% for Montagne Pelée, 32%, for Puy de Dôme, and 17% for Santiaguito). The D-PDC vesicularity distribution is multimodal. In D-PDC deposits highly vesiculated clasts (>40%) are much less abundant in May 8^th^, 1902 of Montagne Pelée (7%) than in P1 eruption (15%) and Puy Chopine (30%).

**Figure 2:**
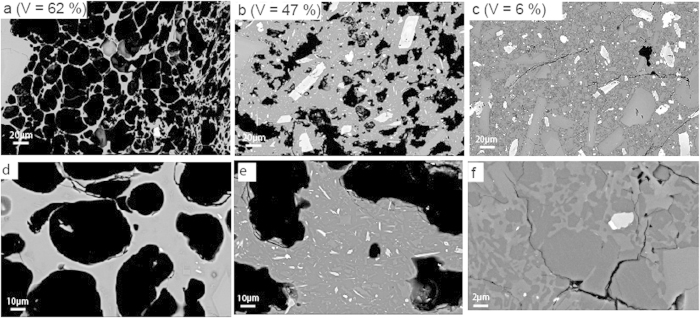
BSE images of representative clasts from Montagne Pelée. Images (**a**–**c**): vesiculated to dense clasts from May, 8^th^, 1902 D-PDC deposit. (**a**) the most vesiculated clasts (bulk rock vesicularity >50%) of D-PDC display characteristic pumiceous textures with subspherical and disconnected vesicles, 10 to 100 μm in diameter, with thin bubble walls (<5 μm) and only rare microlites in glass. (**b**) intermediate clasts have decreasing bulk rock vesicularity, increasing microlite content and bubble wall thickness. (**c**) the densest clasts display heterogeneous textures: most clasts display rare, irregular and small (<20 μm) vesicles with 12 area % cristobalite precipitates. Cristobalite corresponds to the dark grey phase. (**d**) glassy vesiculated clast from May, 8^th^, 1902 D-PDC. (**e**) highly microcrystalline dense clast from 1929 C-PDC. (**f**) cristobalite precipitates - dark grey phase (dense clasts, May, 8^th^, 1902 D-PDC): these are found either as cracked infillings in some large vesicles (up to 30 μm) or as pervasive patches (few μm to 200 μm in size).

**Figure 3:**
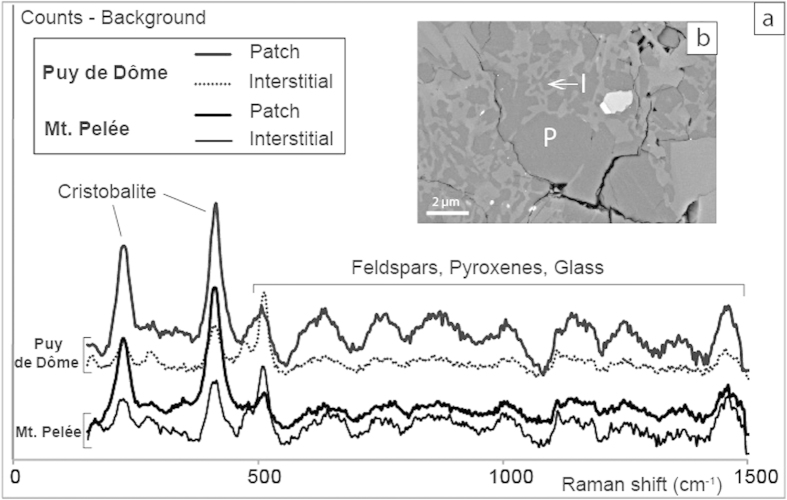
Cristobalite in clasts from Montagne Pelée and Puy de Dôme. (**a**) Raman spectra of crystalline silica patches and of interstitial crystalline silica in dense clasts from Montagne Pelée (May 8^th^, 1902) and Puy de Dôme: both consist of cristobalite (Micro-Raman spectroscopy, Zeiss Supra V55; UPMC, Paris). Y-axis corresponds to counts minus background for the silica precipitates measurements (not to scale). Minor peaks result from the laser excitation of surrounding material (bubble walls) and correspond to the groundmass phases (Feldspars, Pyroxene and glass). Qualitative details are given in the supplementary material. (**b**) BSE image of a dense clast from May, 8^th^, 1902 D-PDC of Montagne Pelée with patches of cristobalite (P) and interstitial cristobalite (I).

**Figure 4:**
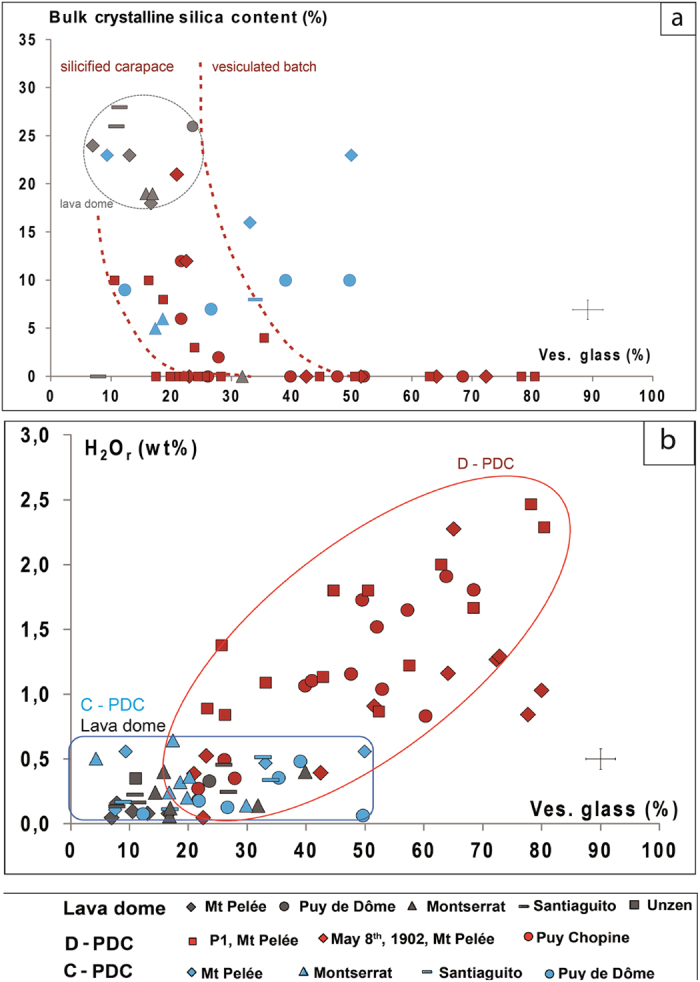
Bulk crystalline silica (cristobalite) and Residual H_2_O content (H_2_O_r_) of samples from lava domes and Pyroclastic Density Current (PDC) deposits, as a function of glass vesicularity (in volume %). Bulk rock vesicularity and H_2_O content are corrected from phenocryst contents to refer to melt, entitled respectively glass vesicularity and residual H_2_O content (H_2_O_r_) (see methodology). (**a**) Cristobalite content vs vesicularity glass. Cristobalite contents are expressed in area % and measured by chemical mapping (see methodology). *Lava domes*. Samples are taken on the different lava domes: Montagne Pelée, Puy de Dôme, Soufrière Hills, Montserrat, Santiaguito. They display a low vesicularity and have the highest content of cristobalite (18–28%). *C-PDCs*. Clasts have a vesicularity from 10 to 50% and are cristobalite-rich with mean cristobalite contents up to ~23% for Montagne Pelée, and ~5–10% for Puy de Dôme, Montserrat and Santiaguito. The cristobalite content is independent of the glass vesicularity. *D-PDCs*. D-PDC samples display a large range of clast types. Beyond a glass vesicularity threshold value of ~40% no or low cristobalite is observed in clasts. Below this threshold, some clasts don’t contain cristobalite whereas others show a negative correlation between vesicularity and cristobalite with a maximum value of 20% (Montagne Pelée). Clasts with glass vesicularities below the threshold, with or without cristobalite, may represent the silicified and rigid lava dome carapace whereas clasts with glass vesicularities above that threshold may represent the inner, less-degassed and vesiculated magma. (**b**) Residual H_2_O content (H_2_O_r_) vs. glass vesicularity. Blue square domain: lava dome and C-PDC samples. Lava dome samples: H_2_O_r_ < 0.4 wt% and V < 40%. C-PDC clasts: H_2_O_r _< 0.7 wt% and V < 50%. Red domain: D-PDC clasts. H_2_O_r_ ranges from 0.2 to 2.5 wt% and glass vesicularity from 10 to 80%. The pre-eruptive H_2_O contents measured on melt inclusions for all these eruptions are significantly higher (>5 wt%^16^,^21^,^22^,^23^).

C-PDC clasts have a narrow and unimodal distribution of vesicularities (20–40%), whereas D-PDC clasts display a much larger range (10–75%) (Fig. 1). The most vesiculated clasts (vesicularity >50%) of D-PDC display characteristic pumiceous textures with subspherical and disconnected vesicles and only rare microlites (Fig. 2a,d). With decreasing vesicularity, vesicle number and size decrease, vesicle shapes become irregular with large vesicles concentrating in some areas, and both groundmass/vesicles and microlite/glass ratios increase (Fig. 2b,c,e). The less-vesiculated clasts show two types of textures: (i) in most clasts, vesicles are rare and crystalline silica precipitates abundant (Fig. 2c,f; Table 1). Crystalline silica exists as cristobalite, as identified by Raman spectrometry (Fig. 3a and supplementary material) and occurs either as cracked infillings in large vesicles (up to 30 μm in diameter) or as a pervasive form in small vesicles (down to 1 μm in diameter) (Figs 2c,f and 3b). The weight fraction of cristobalite in D-PDC clasts decreases with increasing vesicularity (Fig. 4a). No cristobalite is observed in the most vesiculated clasts. (ii) in a few clasts, a texture of sparse, small and irregular vesicles that are widely separated exists; these clasts are cristobalite-free.

Samples from the lava domes and from the C-PDC are poorly- to non-vesiculated (vesicularity < 50%), with vesicle patterns similar to those of the less-vesiculated clasts from the D-PDC. In some samples, coalescence phenomena create numerous irregular channels, sometimes several millimeters long and tens of micrometers wide. Cristobalite is present in all samples collected on lava domes in relatively high proportions (up to 28 area%; Fig. 4a; Table 1). Most C-PDC clasts contain cristobalite (up to 23 area%; Table 1) but its fraction is independent of the vesicularity (Fig. 4a).

### Residual water content

Samples from the lava domes collected as clasts within the C-PDC deposits show low residual H_2_O contents (H_2_O_r_ < 0.6 wt%, bulk rock content corrected for phenocryst content; Table 1). In contrast, clasts collected from D-PDC deposits display larger H_2_O_r_ ranges: 0–1.9 wt% at Montagne Pelée and 0–2.5 wt% at Puy Chopine (Fig. 4b; Table 1). Water content measured on whole-rock samples is considered as pure magmatic water (see methodology).

### Crystal transit time through diffusion profiles in magnetites

**Figure 5:**
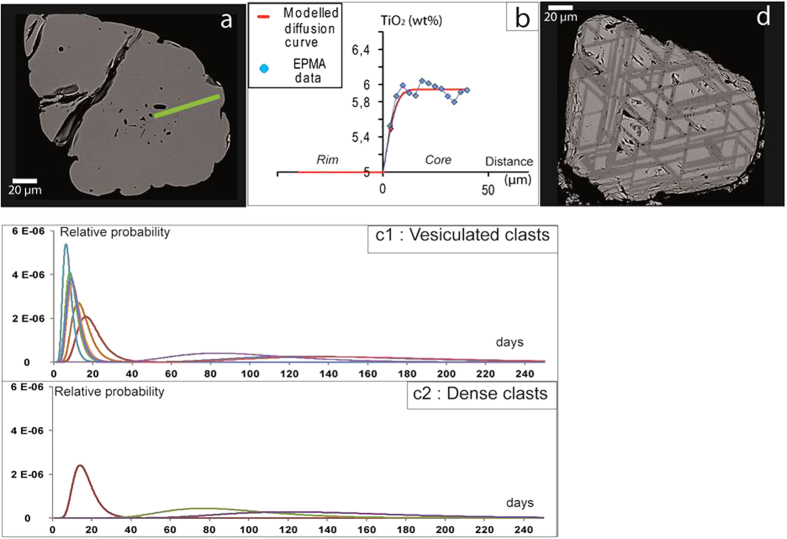
Magnetite textures at Montagne Pelée and Fe-Ti zoning pattern. Samples are from May, 8^th^, 1902 D-PDC (Montagne Pelée). (**a**) BSE image of an unexsolved magnetite (green line: TiO_2_ content profile in Fig. 5 B). The presence of a ring of melt inclusions helps to constrain the profile location. (**b**) Ti-diffusion profile modelling of (A), leading to a short timescale in vesiculated clast (~8 days). (**c**) Relative probability. Residence time data are from titanomagnetite grains recovered from dense and vesiculated pumices c1: Vesiculated clasts leading to “young” timescale (0–30 days) and c2: Dense clasts leading to “old” timescale (30+ days). The graphs show relative probability on an arbitrary vertical scale; populations are scaled by the number of crystals such that the area under the curve should equal unity and can be compared to individual crystal data in that graph. Uncertainties on individual diffusion data points (0.15 log units, 1-sigma) are Gaussian in log-time, meaning that they are asymmetric in linear time, as displayed. The height along the probability axis is scaled in order to conserve area, so that older crystals have a lower peak probability to match their wider uncertainty, and do not dominate signals when combining into population curves. Graph c1 shows the relative clustering of crystals from vesiculated pumices at short timescales. The single short-timescale crystal from dense pumice (c2) plots among the oldest of the crystals from the vesiculated pumice. We infer this to represent a relative increase in ascent velocity for the vesiculated pumice, which is also consistent with the pervasive alteration and exsolution of oxides in the dense pumice. Graphs c1 and c2 show that the older population of crystals in the vesiculated and dense products is effectively coincident and may reflect a similar origin and magma processing pathway. (**d**) BSE image of an exsolved magnetite.

Ti diffusion profiles in magnetite crystals from Montagne Pelée (May, 8^th^ 1902 event) have been studied following the method developed on pyroxenes by Morgan and collaborators^24^. This eruptive event has been chosen to estimate the transit time of magma in the conduit. The textural characteristics of the magnetite crystals depend on the vesicularity of host clasts (Fig. 5a,d). In vesiculated clasts, magnetites display a normal zoning with Ti-rich cores and Ti-poor rims that are in equilibrium with the residual melt (Fig. 5a,b). In dense clasts, two types of textures are evidenced. In some dense, degassed clasts, only few magnetites exhibit the same textural features as vesiculated clasts (~15%); but most crystals are exsolved (Fig. 5d) indicating that they have re-equilibrated at temperatures below the solvus, and likely in oxidizing conditions^25^. In other dense clasts all magnetites are exsolved, indicating a sufficiently long storage time under the appropriate conditions to allow complete exsolution. From over 100 crystals of magnetite separated in vesiculated fragments, 13 diffusion profiles were usable, while over more than 150 separates in dense fragments only three profiles were usable.

## Discussion

Cristobalite is present in clasts from the PDC deposits in highly variable proportions and is heterogeneously distributed, which indicates that precipitation necessarily occurred before the collapse or explosion of the growing lava dome and that cristobalite is not of post-eruptive origin, via hydrothermal circulations or glass devitrification (Fig. 3 and Supplementary material). Cristobalite fills round-shaped voids of any size, characteristic of former gas vesicles (Figs 2f and 4b). The clasts from D-PDC deposits display a large range of textural features from highly degassed, poorly vesiculated and cristobalite-rich to highly vesiculated, poorly degassed and cristobalite-free. Since the D-PDC’s result from explosions at the base of the growing lava dome^7^,^8^, clasts embody a representative sampling of the different zones of the destroyed lava dome, showing a heterogeneous distribution of cristobalite. As cristobalite precipitation is mainly controlled by pressure and temperature decrease^26^,^27^,^28^, it suggests that degassing at atmospheric pressure and low temperature (lower at the lava-dome surface than the 400 °C below which cristobalite precipitates)^28^,^29^ may have produced an outer silicified carapace. The low vesicularity of the outer parts of a lava dome may be due to a combination of three processes during lava dome growth: (i) connection, opening and consequent extreme flattening of the vesicles due to the degassing in open-system during magma ascent at shallow depth in the conduit^9^,^30^, (ii) rapid cooling of the magma at the surface, thus restricting bubble growth^31^, (iii) cristobalite precipitation filling vesicles at low pressure^27^. In addition, slow degassing at shallow depth induces melt crystallization, favoring the development of a rigid carapace delimited by the brittle-ductile transition controlled by melt micro-crystallinity^32^. These processes progressively increase the viscosity and reduce the porosity up to annihilation, creating at the outer surface of the lava dome an impermeable, highly-silicified, mechanically resistant carapace.

**Figure 6:**
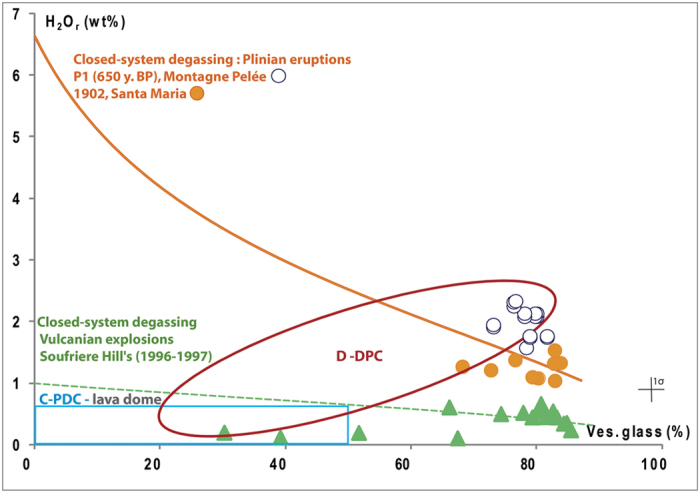
Residual H_2_O content (H_2_O_r_) vs Vesicularity glass of clasts from dome-forming eruptions; comparison with clasts from plinian and vulcanian eruptions. Magmatic H_2_O_r_ is calculated from H_2_O content measured in bulk clast corrected from phenocrysts content (crystallinity). Lava domes, C-PDCs and D-PDCs: blue and red domains as in Fig. 4. Plinian and vulcanian clasts: *Orange circle:* 1902 plinian eruption of Santa Maria (Guatemala;^21^,^22^); *Open circle*: plinian phase of the P1 eruption of Montagne Pelée (650 y. BP; Martinique;^21^,^22^); *Green triangle*: vulcanian phase of Soufrière Hills (Montserrat;^22^). Lines refer to closed system degassing models from initial rhyolitic melts containing 6.5% H_2_O (*orange line*: plinian-type eruptions;^21^,^22^) and 1% of H_2_O (*green dotted line*: vulcanian explosions,^23^) which fit the H_2_O_r_ - vesicularity evolution of clasts. All C-PDC fragments plot below these two closed system evolution lines. D-PDC clasts of every studied eruption span a very large domain between H_2_O-poor, vesicle collapsed clasts and H_2_O-rich, highly vesiculated clasts from plinian eruptions. This extreme heterogeneity indicates that before explosion, the different parts of the lava dome have suffered highly variable degassing conditions varying between pure closed system degassing, likely at the center of the lava dome or as new intruding magma batch, to highly degassed in open system (extreme H_2_O loss and vesicle collapse), likely at the lava dome upper external carapace.

To acquire a significant strength, the carapace must attain a thickness of few meters to several tens of meters in the absence of other consolidating effects^29^. We suggest that silicification may reduce the threshold thickness to the lower range. This impermeable and resistant carapace mechanically isolates the core of the lava dome, thus preventing further volatile exsolution and induced crystallization of the melt confined in this core. The D-PDC’s clasts span a wide range of vesicularity and H_2_O_r_ content, from almost completely degassed, microcrystalline clasts to undegassed, microlite-free clasts (~2.5 wt% of H_2_O_r_ for a glass vesicularity of ~80%; Figs 2d and 4b; Tables 1 and 2). The vesiculated, H_2_O-rich clasts display textural characteristics similar to those of plinian clasts^33^, contrary to the low vesiculated, H_2_O-poor clasts, which are highly microcrystalline and cristobalite-rich. Thus volatile-rich and vesiculated clasts may represent the magma stored within the core of the lava dome and that evolves in a closed system degassing, leading to overpressurization of the upper volcanic edifice (Fig. 6). By contrast, the less-vesiculated, H_2_O-poor clasts may represent magma that evolved in an open-system, degassing in the external part of the lava dome. Overpressures of 0.1–1 MPa are sufficient to surpass the tensile strength of the carapace and to trigger the explosive destruction of the upper part of the lava dome^6^,^34^,^35^. The pressure distribution within the edifice may be significantly modified by the existence of shear stress at the conduit vent^12^: the largest gas overpressure may be located at the conduit wall (horizontal spreading at the vent) or at the center of the conduit (zero horizontal velocity as in the case of a pre-existing lava dome)^36^.

In addition, during magma ascent, as the rheology of the magma is controlled by both volatile behavior and crystal content, the supply system may have a non-linear behavior^6^,^37^. For silicic magmas, the largest part of the magma ascent occurs in a closed system: ascent rate increases with decreasing pressure in response to the decrease in water solubility and to the gas expansion. If, for any reason (variation in wall-rock permeability, wall rock-magma interactions, etc.) the system opens, gas escapes and the ascent rate is dramatically reduced because gas pressure re-equilibrates with the surroundings and melt crystallizes, increasing the bulk magma viscosity^32^. For such slow extrusion rates—which are the case in dome-forming eruptions—magma reaching the surface is generally highly degassed and highly crystalline (85 to 95% solid fraction). In addition lava dome acquire a more fragile behavior that in turn favors fracturing and gas escape. At high extrusion rates, the melt has less time to crystallize during ascent in the conduit, resulting in a more fluid-like behavior^12^. Local shear-induced fragmentation at the conduit walls decoupling the magma column from the wall rock may favor the segregation within the conduit of “small” batches of magma with higher ascent rates, which still evolve in closed-system degassing^10^,^38^. Such magma batches are less degassed and have a higher vesicularity and a lower density. When they intrude the shallow zones of a small, slowly-growing lava dome with a well-formed silicified carapace, they will induce significant pressure buildup under that carapace that may trigger superficial explosion. In addition, the intrusion of less-dense material reduces the load exerted on the conduit and may in turn increase the bulk magma ascent rate^39^. Explosions are laterally-directed because of the lowest strength in the lateral parts of the small lava dome, which has not developed a large talus pile around its base, and large horizontal variations of gas pressure and viscosity gradient that may act as a driving force for lateral gas escape^9^,^11^,^30^.

Diffusion modeling of Ti in magnetites allows estimation of transit time of magma in the conduit. Diffusion profiles are mostly acquired for magnetites from vesiculated clasts as they preserved the diffusive information compared to dense clasts that exhibit generally exsolved magnetites (Fig. 5a,b,d). Results evidence two most likely time intervals depending on the vesicularity of the clasts, a short and a significantly longer interval of time. In vesiculated clasts, Ti-zoning modeling suggests a bimodal distribution of time intervals with peaks centered at ~8 days (n = 6) and ~95 days (n = 4). Three other crystals give timescales below resolution limit (<4 days), suggesting very rapid transit times. In dense clasts, long time intervals (~100 days, n = 2) were found and a single short time interval (~14 days). The long transit times of similar durations found in both vesiculated and dense clasts suggests a synchronous emplacement and common history. By contrast, the short timescales (~8 days, Fig. 5c1, c2) are only evidenced in vesiculated clasts, suggesting a late phase of rapid ascent for these magmas. Most magnetites from dense clasts have undergone exsolution, a reaction to slow cooling and oxidation^25^, on the contrary to magnetites from vesiculated clasts. The single short timescale found in dense clasts (~14 days, n = 1), is twice that of the average found in vesiculated clasts (~8 days, Fig. 5c1, c2). We believe this to be strongly suggestive, if not definitive, of faster ascent rates in the case of vesiculated melt. From these results, we propose a two-step magma ascent process. During dome-forming eruptions magma ascent rates are generally low, favoring open system degassing and leading to the emplacement of dense and highly degassed magmas, with exsolved magnetite crystals. In addition, oxidative exsolution of magnetites is favored by fluid migration related to large development of cristobalite precipitation characteristic of these magmas^25^. In some cases magma batches may ascend more rapidly, at least during the later stages, leading to vesiculated, less-degassed melts. Since the specific textural characteristic of these melts (high vesicularity, low cristallinity, short diffusion times scales and lack of exsolution in magnetites) are preserved, it indicates that the arrival of such magma batches occurs shortly before the superficial explosion and quenching.

**Figure 7:**
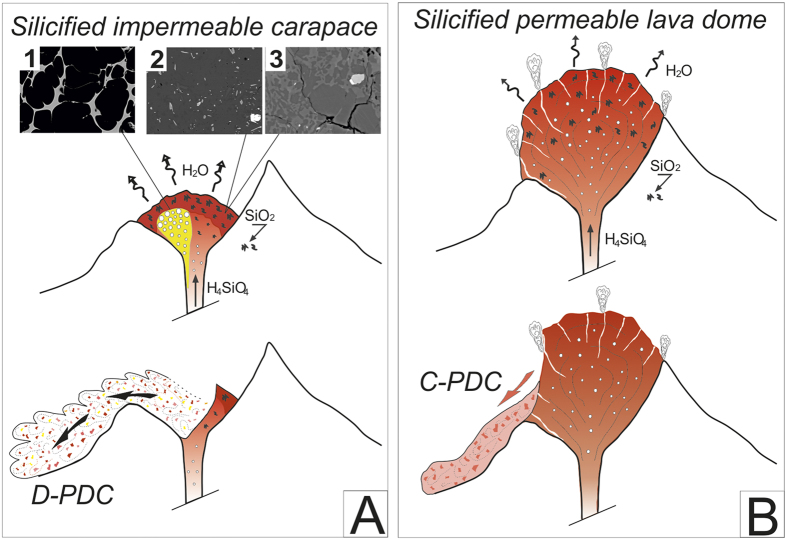
Evolution model for explosive (A) and “non-explosive” (B) dome-forming eruptions. (**A**) In the case of explosive activity, the lava dome is small (less than 0.01 km^3^), possessing a rigid and impermeable silicified carapace formed through H_4_SiO_4_ flux. A small batch of vesiculated magma is isolated by local shear-fragmentation along the conduit wall, and causes a pressure build-up when intruding the growing lava dome. (**B**) In the case of a large lava dome (>to 0.01 km^3^) crystalline silica precipitation occurs pervasively and deep-seated fractures allow magma extrusion without overpressurization of the lava dome. The texture of the lava dome is in that case similar to the dense and silicified part of the explosive lava dome (Fig. 7A, photos 2 and 3). See text for further discussion. The figure was drawn by G. Boudon.

Observations show that the laterally-directed explosions of lava domes occur mainly at the onset of the eruption when the lava dome has still a small size (Montagne Pelée, 1902^7^,^8^, 650 BP^16^, Puy Chopine^20^). On the basis of the volume of the D-PDC deposits, we can estimate that for these eruptions, the volume of the lava domes before explosion was probably not greater than 0.01 km^3^. Two factors may explain these observations.

i/the load of a small lava dome exerts a low pressure on its basement and the upper parts of the feeding conduit: the pressure buildup necessary to generate explosion is therefore low^21^,^37^. Possibly, exsolved volatiles may migrate from ascending magma and concentrate within erupting magma beneath the impermeable carapace, leading to overpressurization^37^. The low proportion of vesiculated clasts (vesicularity >40%) in the D-PDC (Fig. 1) indicates that only a small batch of vesiculated magma is at the origin of the pressure buildup (Fig. 7A). The fraction of vesiculated clasts in lava dome explosion products is much lower in the May 8^th^ 1902 eruption of Montagne Pelée (7%) than in the P1 eruption (15%) and Puy Chopine eruption (30%). These variations likely reflect variations in the depth of the explosion or the size of the lava dome. An explosion occurring within the lava dome requires less energy and likely a lower volume of vesiculated, less-degassed magma than an explosion occurring at the base of a lava dome or in the upper part of the feeding conduit. Lava dome growth is a self-defeating process: when the lava dome grows, the load progressively increases and the overpressure generated by exsolved gas becomes insufficient to overcome the load pressure and the lava dome strength to trigger an explosion^39^.

ii/the size of the growing lava dome also controls cristobalite precipitation efficiency. To generate an impermeable, resistant carapace through cristobalite precipitation, the flux of silica-rich fluids has to be large relative to the lava dome size (Fig. 7B). These fluids are generated over the whole H_2_O-saturated magma column and percolate through the lava dome at the surface within which they precipitate cristobalite^27^. For a given flux of fluids corresponding to a given magma extrusion rate, the mean thickness of the silicified carapace decreases as the volume of the lava dome increases. In addition, a voluminous lava dome doesn’t favor the permanence of a continuous impermeable carapace due to development of deep-seated fractures intersecting the outer parts of the lava dome. A low carapace strength less efficiently maintains overpressure inside the lava dome and degassing may occur passively through deep-seated fractures, reducing its explosive potential (Fig. 7b).

In recent decades, lava dome-forming eruptions have exhibited various type of explosivity. During the November 2010 eruptive phase of Merapi (Indonesia), explosive destruction of the emerging lava dome occurred one week after the beginning of its growth^40^, likely due to the ascent of an undegassed batch of magma^38^. Mount Lamington (Papua New Guinea) experienced similar explosive activity that destroyed the growing lava dome in the first days of the 1951 eruption^41^. In the case of cryptodome emplacement (Mt St. Helens, 1980 or Bezimianny, 1956), similar features have been described in clasts sampled in the blast deposits, with the coexistence of vesiculated clasts, with high H_2_O_r_ content (up to 1.5 wt%) and dense clasts which are highly crystalline, silica-rich and highly-degassed^42^. The presence of crystalline silica precipitates in the rocks surrounding the cryptodome likely favoured the isolation of a less-degassed batch of magma^43^. It is thought, however, that the triggering factor of the explosion was the large decompression induced by the flank-collapse, because the overpressure reached inside the small cryptodome was likely insufficient to trigger it alone^6^. Similarly, large dome collapse leading to a sudden depressurization of the magma column may trigger fragmentation deeper in the conduit and produce vulcanian explosions, as at Soufrière Hills (Montserrat) or at Mount Unzen (Japan)^1^,^3^,^5^. These vulcanian products have a higher vesicularity and a lower H_2_O content (<0.6 or 0.7 wt% H_2_Or^23^, Fig. 6) than the vesiculated clasts from the D-PDCs and represent fragmentation conditions (pressure and water content) of magmas quenched a few hundred meters deep in the conduit.

Lava dome eruptions are highly controlled by shallow H_2_O degassing processes and silica precipitation. Magma silicification (by cristobalite precipitation) and densification (by flattening of the vesicles) are systematic processes during dome-forming eruptions. But the conditions able to generate sufficiently large impermeable carapace at the periphery of a growing lava dome, allowing a possible explosion caused by the overpressure generated by the intrusion of a batch of undegassed magma, are rarely achieved. Laterally directed explosions at the base of a growing lava dome or in the superficial part of the conduit may occur when two conditions are simultaneously fulfilled: a small size of the lava dome and a significant precipitation of vapor phase silica in voids and vesicles creating an impermeable carapace that prevents further gas loss and allows local overpressurization. Estimation of initial sizes of destroyed lava dome is difficult and can only be performed using well documented lava-dome eruptions such as the Montagne Pelée ones and through estimation of the volume of the D-PDC’s. The proposed model for superficial explosivity of lava domes is based on the combination of textural, geochemical and petrological criteria that are obtained through a systematic study of a very detailed sampling of deposits resulting from both superficial explosions and gravitational dome collapses: Puy Chopine, Montagne Pelee, Montserrat and Santiaguito. In the latter two cases sampling was limited by access concerns due to current eruptive activities. These eruptions also cover a large range of magma compositions: from andesitic to trachytic. We believe that the resulting model is applicable to a variety of volcanic systems worldwide and rhyolitic melt compositions, although testing is necessary for other melt compositions.

Superficial, lava dome explosion-generating D-PDC’s, demonstrably devastating events, embody one of the most hazardous aspects of dome-forming eruptions. Even if this type of event is less predictable because it dominantly occurs at the onset of such eruptions, and because no method exists to reliably detect potential overpressurization within a growing lava dome, the possibility of such activity must be taken into account during the management of future volcanic crises, especially during the early stage of dome-forming eruptions. This type of volcanic activity is hazardous regarding both pyroclastic activity and the effects of crystalline silica-rich volcanic ash dispersed into the atmosphere^44^.

## Methodology

### Sampling

For all the eruptions, the lava dome was sampled. For the dilute pyroclastic density currents (May 8^th^, 1902 and P1 eruption of Montagne Pelée, Puy Chopine in La Chaîne des Puys), and for the concentrated pyroclastic denstity currents (1929 eruption from Montagne Pelée (2 deposits sampled), Puy de Dome eruption, 1929 eruption of Santiaguito), we collected at least 100 clasts for each deposit in a fraction 16–32 mm, to have a statistical representation of the density distribution. In the concentrated pyroclastic density currents from the recent eruption of Soufrière Hills, Montserrat, only few clasts were sampled in the deposits.

### Density and Vesicularity of clasts

Densities are measured on fragments taken on the lava domes and at least one hundred clasts from each associated PDC deposits by the “3 weighing method” (weighing uncoated clasts in air, then paraffin coated clast in air and in water). The bulk rock vesicularity is calculated from density measurements using the Dense Rock Equivalent density and corrected for phenocryst content to refer to the initial melt (glass vesicularity). The phenocryst weight fraction is estimated by point counting (Puy de Dôme and Puy Chopine samples^45^) or by mass balance calculations on separate minerals and matrix using major and trace element data^16^.

### Residual H_2_O content

Water contents are measured by H_2_ manometry (adapted from 46) on bulk samples spanning the whole density range of each eruption. Data are then corrected for phenocryst content to obtain the residual H_2_O content of glass or matrix (H_2_O_r_). Water measured is considered as pure magmatic water on selected samples, as ensured by step-heating during extraction^47^. In addition, textural investigations never evidenced rehydration processes (Fig. 2). δD composition measurements of lava dome fragments and pumice clasts from the P1 eruption exclude significant contribution of meteoric water in this material^16^. Similar isotopic data on obsidian clasts from fallout deposits, lava domes and flows from the Western United States show that total water content is controlled by degassing processes in such samples of similar composition^48^,^49^.

### Textures (vesicularity and microcrystallinity) and crystalline silica (cristobalite) contents

They were determined using SEM images and chemical mapping by Energy Dispersive X-ray Spectrometry (EDS) acquired with a Zeiss Supra V55 equipped with a Brucker Silicon drift Detector (UPMC, Paris)^33^. Chemical mapping (2 to 5 maps per sample) of major elements (Si, Ti, Al, Fe, Mg, Ca, Na, K, P, Cl) at magnification 100× and 300× are used to estimate the contents in residual glass and crystals (phenocrysts + microlites) and cristobalite precipitates (Si maps). The mean relative standard deviation is ~15 area % for cristobalite contents.

### Diffusion modelling

Diffusion chronometry estimates the time elapsed since the onset of a disequilibrium, for example when crystals are rapidly moved to an environment with different physical or chemical conditions (T, P, fO_2_, composition), or when they grow rims that are not in equilibrium with their cores. The existence of partial, diffusion-mediated re-equilibration allows the timing of such particular phenomena to be determined by measuring the extent and lengthscale of re-equilibration. The rate at which diffusion occurs is strongly dependent on temperature, therefore when the crystal cools these compositional gradients can become “frozen in”. Modeling the measured composition (elements or isotopes) gradients allows estimation of the time between the last disequilibrium event and quenching. Such modeling using Fick’s diffusion laws requires the knowledge of the diffusion coefficients and composition measurements at high spatial resolution and analytical precision. Changes in temperature, pressure and volatile content of the environment of crystals may significantly affect the growth and composition of volcanic minerals such as magnetites, feldspars and pyroxenes^24^,^25^,^50^ (Fig. 5a,b). Here the methodology developed for pyroxenes by Morgan on the examples of Oranaui eruption (New Zeland)^24^ is adapted for magnetites. Ti zoning in magnetite is essentially temperature-dependent but may also be affected by sub-solidus exsolution (Fig. 5d). If magnetites are unexsolved, Ti content may be used to constrain T and/or fO_2_ changes during the late stages of magma ascent in the conduit and magnetite crystallisation, shortly before quenching by explosion (timescales of the order of days to months,^50^). At Montagne Pelée, vesiculated (V > 50%) and dense (V < 30%) clasts were crushed and the powder sieved. Magnetites were separated by hand-picking in the fraction 250–315 μm. Ti profiles of unexsolved magnetites were obtained using first SEM images to identify the best candidates for diffusion modeling. Ti zoning in crystals is difficult to recognize on the basis of SEM images or chemical mapping due to the low contrast in composition, but the occurrence of a ring of melt inclusions helps identification of suitable crystals (Fig. 5a). Then, quantitative Ti profiles were performed, together with major elements (Si, Ti, Al, Fe, Mn, Mg, Ca, Na, K, Co) by electron microprobe (Cameca, SX-Five, Camparis, UPMC, France). Analyses are performed at 15 keV and 20 nA with a focused beam of 3 μm. We used counting time of 10s for all elements except Ti(60s). Diffusion profiles are between 40 and 120 μm long, from core to rim with a 5 μm step. Then Ti diffusion profile may be fit by diffusion models at 875–900 °C and fO_2_ of NNO+0.4–0.8^51^ using the diffusion data published giving a diffusion coefficient of 2.22854 10^−17^ m^2^/s^52^. Uncertainty is estimated by combining uncertainties in diffusion temperature and oxygen fugacity, and propagating this onto the timescale; the uncertainties are expressed as Gaussian in log-time, with a 1-sigma uncertainty value of ~0.15 log units, meaning that they display asymmetrically in linear time as shown in Fig. 5.

## Additional Information

**How to cite this article**: Boudon, G. *et al*. What factors control superficial lava dome explosivity? *Sci. Rep*. **5**, 14551; doi: 10.1038/srep14551 (2015).

## Supplementary Material

Supplementary Information

## Figures and Tables

**Table 1:** Textural and geochemical data, from Montagne Pelée (Martinique), Chaîne des Puys, Santiaguito (Guatemala) and Soufrière Hills (Montserrat).

	d measured kg.m^−3^	Ves WR %	Ves glass %	H_2_O WR wt%	H_2_O r wt%	Silica area %		d measured kg.m^−3^	Ves WR %	Ves glass %	H_2_O WR wt%	H_2_O r wt%	Silica area %
Montagne Pelée, Martinique													
*P1, D-PDC*							*1902, Lava dome*						
ME 1311fy 23	0,88	67	80	1,03	2,29	0	MH 804a	2,30	13	17	0,05	0,08	18
ME 1311fy 9	1,46	45	63	0,9	2,00	0	MG 804	2,37	11	13	0,05	0,08	23
ME 1311fy 2	2,1	23	35			4	MG 802	2,49	6	7	0,03	0,05	24
ME 1311fy 29	2,3	18	28			0							
ME 1311fy 20	2,32	13	26	0,62	1,38	0	*May 8*^*th*^*1902, D-PDC*						
ME 1311fy 30	2,3	15	24			0	ME 1314-1	2,31	13	21	0,21	0,39	21
ME 1311fy 14	2,4	13	21			0	ME 1314-2	1,86	30	42	0,21	0,39	0
ME 1311fy 6	2,4	12	19			8	ME 1314-3	2,28	14	22	0,03	0,05	12
ME 1311fy 18	2,5	11	17			0	ME 1314-4	1,64	38	52	0,49	0,91	0
ME 1311fy 32	2,5	10	16			10	ME 1314-5	1,05	60	72	0,68	1,27	0
ME 1311fy 10	2,6	7	11			10	ME 1314-6	1,30	51	64	0,62	1,16	0
ME 1311fx 6	2,4	12	20			0	ME 1314-7	2,27	14	23	0,28	0,52	0
ME 1311fx 25	2,4	14	22			0	ME 1314 y1 6	0,87	67	78	0,45	0,84	n.d.
ME 1311fx 30	0,97	64	78	1,11	2,47	0	ME 1314 y1 10	0,80	70	80	0,55	1,03	n.d.
ME 1311fx 10	1,79	32	50	0,81	1,80		ME 1314 y1 11	1,03	61	73	0,69	1,29	n.d.
ME 1311fx 8	1,93	27	45	0,81	1,80		ME 1314 y1 12	1,27	52	65	1,215	2,28	n.d.
ME 1311fx 12	2,35	15	24			3							
ME 1311fx 21	2,44	12	19			8	*1929, Lava dome*						
1311E	1,75	34	52	0,39	0,87		MG 901	2,42	9	10	0,06	0,10	n.d.
1311F				0,28	0,62		MG 803	2,47	7	8	0,10	0,16	n.d.
1311F1	2,31	13	26	0,38	0,84								
1311F3	1,61	39	57	0,55	1,22		*1929, C-PDC*						
901F1	2,36	11	23	0,4	0,89		ME 1317-1	2,53	4	8	0,06	0,11	n.d.
901F2	1,97	26	43	0,51	1,13		ME 1317-2	2,04	23	33	0,26	0,47	16
901F3	2,20	17	32				ME 1317-3	1,65	38	50	0,31	0,56	23
901F4	2,18	18	33	0,49	1,09	5	ME 1317-4	2,50	6	9	0,31	0,56	23
French Massif Central													
Puy Chopine							Puy de Dôme						
*D-PDC*							*C PDC*						
PC- 139	0,92	64	68	1,39	1,81	0,0	PdD 56	1,74	35	35	0,28	0,35	n.d.
PC- 133	1,05	59	64	1,47	1,91	n.d.	PdD 84	2,05	23	22	0,14	0,18	n.d.
PC- 91	1,14	56	60	0,64	0,83	n.d.	PdD 41	1,94	27	27	0,1	0,13	7
PC- 130	1,22	52	57	1,27	1,65	n.d.	PdD 10	2,26	15	12	0,06	0,08	9
PC- 19	1,35	47	52	1,17	1,52	0,0	PdD 44	1,65	38	39	0,38	0,48	10
PC- 11	1,33	48	53	0,8	1,04	n.d.	PdD 80	1,39	48	50	0,05	0,06	10
PC- 6	1,41	45	49	1,33	1,73	n.d.							
PC- 99	1,46	43	48	0,89	1,16	0,0	*Lava dome*						
PC- 58	1,65	36	40	0,82	1,06	0,0	PdD	2,01	24	24	0,26	0,33	26
PC- 29	1,62	37	41	0,85	1,10	n.d.							
PC- 50	1,97	23	26	0,38	0,49	0,0							
PC- 70	1,93	25	28	0,27	0,35	2,0							
PC- 68	2,07	19	22	0,14	0,18	12,0							
PC- 56	2,07	19	22	0,21	0,27	6,0							
*lava dome*													
PC	2,1	18				n.d.							
Santiaguito (Guatemala)							Soufriere Hills (Montserrat)						
*Lava dome*							*Lava dome*						
							Castle Peak						
SM10a	2,44	8	8	0,1	0,13	0	MO1	2,44	11	16	0,2	0,40	n.d.
SM11	2,05	23	27	0,2	0,25	n.d.	MO2	2,44	11	16	0,2	0,40	19
SM12	2,38	10	11	0,2	0,22	26							
SM13	2,37	11	11	0,1	0,16	28	1995–1996						
SM15	2,07	22	26	0,3	0,46	n.d.	MVO24	2,42	12	17	0,03	0,06	n.d.
							MVO25	2,42	12	17	0,06	0,12	19
*1929, C-PDC*							MVO26	2,47	10	14	0,12	0,24	n.d.
SM9 3c1	1,92	28	33	0,4	0,51	n.d.	MVO27	2,14	22	32	0,07	0,14	0
SM9 3c3	2,42	9	9	0,1	0,17	n.d.	182a	1,97	28	40	0,2	0,40	n.d.
SM9 3c7	2,26	15	17	0,1	0,11	n.d.							
SM8	1,89	29	34	0,2	0,34	8,0	*1995-1996, C-PDC*						
							Mo96-2	2,42	12	17	0,12	0,24	n.d.
							Mo96-3C3	2,41	12	17	0,32	0,64	5
							Mo96-3C6	2,39	13	19	0,16	0,32	6
							96-02A RT	2,37	14	20	0,1	0,20	n.d.
							96-01A	2,18	21	30	0,07	0,14	n.d.
							96-11B	2,36	14	20	0,18	0,36	n.d.
							96-11C	2,63	4	4	0,25	0,50	n.d.
							96-11C-Cb	2,63	4	4	0,25	0,50	n.d.

**d measured (kg.m**^**−3**^): the density is obtained by the “3 weights” methods (weighing uncoated clasts in air, then paraffin coated clast in air and in water) for clasts spanning the 16–32 mm sieve size fractions.

**Ves WR (%):** the whole rock vesicularity is calculated from density measurements using the Dense Rock Equivalent density. DRE density is specified for each system below the data; the DRE density is obtained by pycnometry measurement on fine-grained whole-rock powder on a minimum of 3 samples for each system.

**Ves glass (%):** The vesicularity is calculated from density measurements using the Dense Rock Equivalent density and corrected from phenocrysts content to refer to the initial melt. The crystallinity for each system is reported below the data. Ves_glass_ is calculated from Vg/Vl by the formula: Ves_glass_ = (Vg/Vl)/(1+Vg/Vl). The last two parameters are best suited for tracing the effect of degassing, because they only depend on initial melt content which is the only source of exsolved fluid (H_2_O) and do not depend on the initial cristallinity of the magma which is highly variable in the different studied eruptions.

**H**_**2**_**O WR (wt%):** obtained by H_2_ manometry^46^.

**H**_**2**_**O**_**r**_**(wt%):** residual H_2_O content calculated from H_2_O WR corrected from phenocryst content.

**Silica (area %):**obtained by chemical mapping (2 to 5 per sample) at magnification 100x of Si. The mean relative standard deviation is <5% for crystalline silica contents.

**Table 2:** Dense Rock Equivalent density for studied magmas and the cristallinity (wt%).

		DRE	Cristallinity (wt%)
Montagne Pelée, Martinique	P1, D-PDC	2,65	55
	1902 D-PDC	2,65	50
	1902 lava dome	2,65	40
	1929 C-PDC	2,65	40
	1929 lava dome	2,65	40
Chaîne des Puys (France)	Puy Chopine	2,56	23
	Puy de Dôme	2,66	21
Santiaguito (Guatemala)	1929, C PDC	2,65	29
	Lava dome	2,65	33
Montserrat	Lava dome	2,75	50
	C PDC	2,75	48
