# Supplementary material for: What factors control superficial lava dome explosivity?
Source: Sci Rep. 2015 Sep 30;5:14551. doi: 10.1038/srep14551 (PMC4588564; doi:10.1038/srep14551)
Supplement: Supplementary Information [file srep14551-s1.pdf]

What factors control the superficial lava dome explosivity?

Georges Boudon[1*], Hélène Balcone-Boissard[2,3], Benoît Villemant[2,3], Daniel J. Morgan[4]

[1] *Institut de Physique du Globe de Paris, Sorbonne Paris Cité, Université Paris Diderot, UMR 7154 CNRS,*

1, rue Jussieu, 75238 Paris, France

[2] *Sorbonne Universités, UPMC Univ. Paris 06, UMR 7193, ISTEP, F-75005, Paris, France.*

[3]*CNRS, UMR 7193, ISTEP, F- 75005, Paris, France*

[4]*Institute of Geophysics and Tectonics, School of Earth & Environment, University of Leeds, Leeds, LS2 9JT UK.*

* corresponding author

Supplementary information

Figure 1:

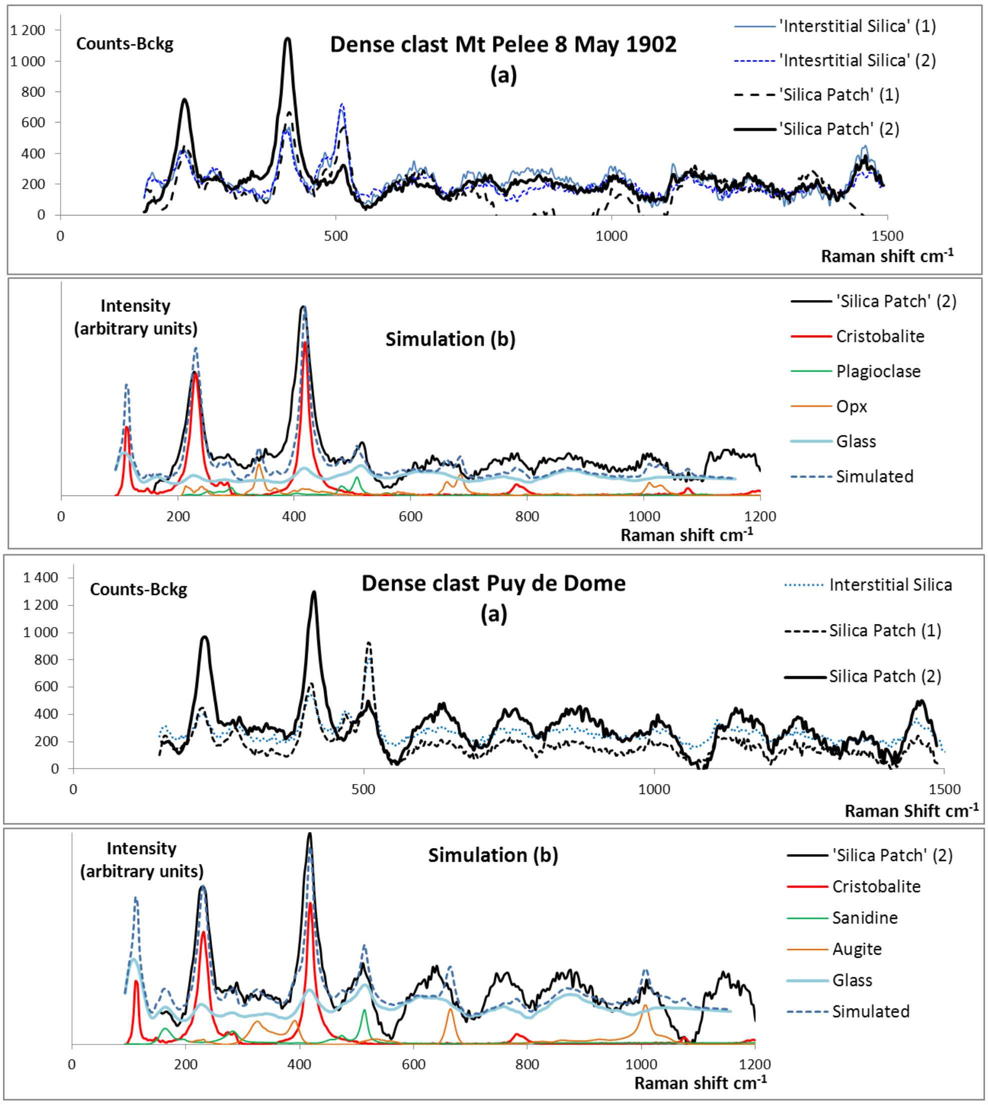

Figure 1: (a) Raman spectra of cristobalite precipitates in Montagne Pelée and Puy de Dôme clasts. (b) interpretation using Raman spectra of minerals (RRUFF database; Downs, 2006) and of a microlite free rhyolitic glass (Puy Chopine). All spectra are similar with dominant cristobalite and minor contributions of surrounding groundmass glass and minerals: Feldspars, Pyroxenes (Montagne Pelée: plagioclase and orthopyroxene; Puy de Dôme: alkali feldspar and clinopyroxene). Groundmass phase contributions are larger for pervasive crystalline silica precipitates because of their much lower size and the unavoidable excitation of surrounding material. Y-axis corresponds to counts minus background for the cristobalite precipitates measurements (not to scale). Minerals and glass spectra intensities are arbitrarily adjusted to provide a simulated spectra best-fitting the measured spectra.

Reference: Downs R. T., 2006, The RRUFF Project: an integrated study of the chemistry, crystallography, Raman and infrared spectroscopy of minerals. Program and Abstracts of the 19th General Meeting of the International Mineralogical Association in Kobe, Japan, O03-13.
